# Supplementary material for: Members of the ELMOD protein family specify formation of distinct aperture domains on the Arabidopsis pollen surface
Source: eLife. 2021 Sep 30;10:e71061. doi: 10.7554/eLife.71061 (PMC8483735; doi:10.7554/eLife.71061)
Supplement: Supplementary file 1. [file elife-71061-supp1.docx]

**Supplementary File 1.** **Primers, molecular markers, and mutants/transgenic lines used in this study.**

**Supplementary File 1A.** Primers used in this study.

| **Primer name** | **Sequence (5’-3’)** | **Purpose/Details** |
| --- | --- | --- |
| Primers for genotyping | | |
| *mcr*-F | CAGGTTGGGGATCGGTCTGTGTGGGAATCC | *mcr-1* genotyping*.*  *(mcr-1* amplicon is cut with *Mnl*I; run on 4% gel) |
| *mcr*-R | GGTAGGTAAGTAAATTCATG |  |
| *elmod_c*_LP | TCCTTTGAATTGTATCGGTGG | Genotyping for T-DNA insertions in *ELMOD_C* through *ELMOD_F*.  (WT, LP+RP; mutant, LBb1.3+RP) |
| *elmod_c*_RP | ATGGATCTTTTCCTTGCCATC |  |
| *elmod_d*_LP | CAAGAGAGAGACCAGCTAGGG |  |
| *elmod_d*_RP | ACATGTGTTTGCTTTAACCGC |  |
| *elmod_e*_LP | AGGAAAGACTTCAAAGGCTGC |  |
| *elmod_e*_RP | GCTCTCTCTCCAGCTGATTCC |  |
| *elmod_f*_LP | TGATGTAGATGGGAGGAA |  |
| *elmod_f*_RP | CTTAGAGGCACCAAGCAG |  |
| LBb1.3 | ATTTTGCCGATTTCGGAAC |  |
| TES-SALK_073961-LP | AGTCAGAGTTAAGAAGCCCGG | Genotyping *tes* (SALK113909_C) in three-primer reaction with LBb1.3 |
| TES-SALK_073961-RP | TCCCAGAAAGAGGTGAACATG |  |
| Atrec8-3-N836037U | CTCATATTCACGGTGCTCCC | *atrec8-3* genotyping.  (WT, Atrec8-3-N836037U  + Atrec8-3-N836037L; mutant, LB3Sail + Atrec8-3-N836037L) |
| Atrec8-3-N836037L | GGGGGAAAAGAGAAAGGTTC |  |
| LB3Sail | TAGCATCTGAATTTCATAACCAATCTCGATACAC |  |
| osd1-3-Fw | CTCATTGTCTCTCTACACAGTTTTGGAGTT | *osd1-3* genotyping. (WT, osd1-3-Fw + osd1-3-Rev; mutant, T-DNA-Fw+osd1-3-Rev) |
| osd1-3-Rev | TCGCATATGGTCTCTTCTCCTAAGTAAAGA |  |
| T-DNA-Fw | CTGGGAATGGCGAAATCAAGGCATC |  |
| Atspo11-1-3-N646172U | AATCGGTGAGTCAGGTTTCAG | *atspo11-1-3* genotyping.  (WT, Atspo11-1-3-N646172U  + Atspo11-1-3-N646172L;  mutant, LBSalk2  + Atspo11-1-3-N646172L) |
| Atspo11-1-3-N646172L | CCATGGATGAAAGCGATTTAG |  |
| LBSalk2 | GCTTTCTTCCCTTCCTTTCTC |  |
| cenh3-1-F | GGTGCGATTTCTCCAGCAGTAAAAATC | *cenh3-1* genotyping. (Wild- type amplicon is cut with *Eco*RV). |
| cenh3-1-R | CTGAGAAGATGAAGCACCGGCGATAT |  |
| Cas9-F | CACGACATTGATTACAAGGATGATG | Genotyping for presence of the CRISPR/Cas9 constructs |
| Cas9-R | CTCCTCGAAGAGCTGGTTGTAC |  |
| At4g22600-DF | CCATTTAGACAAGGGCTTG | *inp1-1* genotyping.  (*inp1-1* amplicon is cut with *Sac*I) |
| At4g22600-DR | AACTTGATACGACGAGACC |  |
| At1g15320-BF | GAGAGATCTCACCAACACGAATC | *inp2-1* genotyping.  (*inp2-1* amplicon is cut with *Acc*I) |
| AD402 | CGCAATGCTGTTCTGTTCTTGCGTC |  |
| AD239 | TGTTTCTGCAACTTTTACCGG | *d6pkl3-2* genotyping.  (WT, AD239+AD285; mutant, AD238+LBa1) |
| AD285 | CAAACAAGACAGCAAAAAGATC |  |
| AD238 | TAACAAGCTTCTTCCTCGCTG |  |
| LBa1 | TGGTTCACGTAGTGGGCCATC |  |
| Primers for CRISPR/Cas9 mutagenesis | | |
| *elmod_a* sgRNA-F | ATTGGAGGAAAATGGTACCTGAGC | Cloning sgRNA for *ELMOD_A* |
| *elmod_a* sgRNA-R | AAACGCTCAGGTACCATTTTCCTC |  |
| *elmod_e* sgRNA-F | ATTGACAAAGAGCCGCGATGGCAG | Cloning sgRNA for *ELMOD_E* |
| *elmod_e* sgRNA-R | AAACCTGCCATCGCGGCTCTTTGT |  |
| Primers for cloning | | |
| MCRpr-F | ACAAAAGCTGGAGCTCATGAAGAAATGGTGATTGCATCG | Cloning *MCR* promoter |
| MCRpr-R | TATCATCCATACCGGTCTCTCACTTTCTCTTATTCGC |  |
| MCR-*Age*I-F | AAGTGAGAGACCGGTATGGATGATAGAGAAGGCTCGTT | Cloning *MCR* genomic DNA and *MCR* CDS  Cloning *MCR* genomic DNA without stop codon |
| MCR-*Nco*I-R | CCTTGCTCACCATGGCTTGATTGAGAAGAGCGTAGGAGG |  |
| MCR-stop-*Nco*I-R | GGAACCATGGCTATTGATTGAGAAGAGCGTAGGAG | Cloning *MCR* CDS with stop codon |
| MCR-798-*Nco*I-R | GGAACCATGGGTAATAAGCTACGTGGTGGAC | Cloning MCR genomic DNA with 798-bp downstream of stop codon |
| MCR-3UTR-F | AAGTAAAGCGGCCGCCCCTTATGATGATATTAAATAG | Cloning 3’UTR of *MCR* |
| MCR-3UTR-R | CGGACTAGTTCTAGAGCTTGTGAAGGCCGTCTCTA |  |
| EApr-F | ACAAAAGCTGGAGCTCGAGTGGAGGACAGGACATAG | Cloning *ELMOD_A* promoter |
| EApr-R | ATCATCCATGGATCCCTCTCACTCTCTTCCGAGTCAC |  |
| EA-*Bam*H I-F | ggatccATGGATGATAGAGGAGGATCG | Cloning *ELMOD_A* genomic DNA |
| EA-*Nco*I-R | CCCTTGCTCACCATGGCTTGACTGAGAAGACTGTATG | Cloning *ELMOD_A* genomic DNA without stop codon |
| EA-298-*Nco*I-R | CCCTTGCTCACCATGGCCAAATGATTTATTAGCCCAAAACAC | Cloning *ELMOD_A* genomic DNA with 3’UTR |
| ELMOD_C-*Age*I-F | AAGTGAGAGACCGGTATGCGAGTTGTCAGAATATCATG | Cloning *ELMOD_C* genomic DNA without stop codon |
| ELMOD_C-*Nco*I-R | CCTTGCTCACCATGGCAGAGAGAAGAGAGAAAGAAGGCA |  |
| ELMOD_D-*Age*I-F | AAGTGAGAGACCGGTATGCTTTCGTGTAATTCTCTAGG | Cloning *ELMOD_D* genomic DNA without stop codon |
| ELMOD_D-*Nco*I-R | CCTTGCTCACCATGGCAGCTGCCCCTTGTACATCTA |  |
| ELMOD_E-*Age*I-F | AAGTGAGAGACCGGTATGAAGCGTGGGAAAGGC | Cloning *ELMOD_E* genomic DNA without stop codon |
| ELMOD_E-*Nco*I-R | CCTTGCTCACCATGGCTTGGAACAAGAGATTGTAGGCAG |  |
| ELMOD_F-*Age*I-F | AAGTGAGAGACCGGTATGGCATCTGCGACTCTGAG | Cloning *ELMOD_F* genomic DNA without stop codon |
| ELMOD_F-*Nco*I-R | CCTTGCTCACCATGGCGTACAAAAGATTGAAAGCAGGC |  |
| ELMOD_Epr-F | ACAAAAGCTGGAGCTCGATCTGATCACCGTTGATAGG | Cloning *ELMOD_E* promoter |
| ELMOD_Epr-R | CACGCTTCATACCGGTCCTCCTCTACCTGCAAAC |  |
| Primers for PCR-based site-directed mutagenesis | | |
| MCR-R127K-F | AGATTTTAAGTATAACACGGACTTTCCTTATCATT | Creating *gMCR^R127K^* |
| MCR-R127K-R | GTGTTATACTTAAAATCTGTTGACGGATCTTTGC |  |
| ELMOD_A-R127K-F | TTTTAAGTAACGTCTCATCTCATTGTCA | Creating *gELMOD_A^R127K^* |
| ELMOD_A-R127K-R | GAGACGTTACTTAAAATCTGTCGATGGATCTTTTCC |  |
| MCR-D121N-F | GGTTGGCAGGGCAAAAATCCGTCAACAGATTTTAGGTATAACAC | Creating *gMCR^D121N^* and *gMCR^D121N/G129C^* |
| MCR-D121N-R | TTTTGCCCTGCCAACCCATCTC |  |
| MCR-G129C-F | TCATCTGGCAGGGGTTGTGGTTTCATATCTCTTGAAAACTTGC | Creating *gMCR^G129C^* and *gMCR^D121N/G129C^* |
| MCR- G129C-R | AACCCCTGCCAGATGATGCACT |  |
| ELMOD_E-N121D-F | GGATGGCAAGGTCCCGATCCATCTACCGATTTCAGGTAAG | Creating *gELMOD_E^N121D^* and *gELMOD_E^N121D/C129G^* |
| ELMOD_E-N121D-R | CGGGACCTTGCCATCCCATTTC |  |
| ELMOD_E-C129G-F | TTGTTTTGTAGGGGTGGCGGATTTATCGCTCTAGAAAATTTAC | Creating *gELMOD_E^C129G^* and *gELMOD_E^N121D/C129G^* |
| ELMOD_E-C129G-R | CACCCCTACAAAACAAATGTTTAACAAA |  |

**Supplementary File 1B.** Molecular markers used for mapping in this study.

| **Name** | **Chr.** | **Position on chromosome (AGI)** | **Primers (5’ to 3’)** |
| --- | --- | --- | --- |
| Mt0-1-9Mb | I | 9387879 | F: TACTCGAGCAACTAACTATG |
|  |  |  | R: AGTCCTCTTACCACAATCTT |
| Mt0-1-13Mb | I | 13462876 | F: TGCATGACAGCACGAGAC |
|  |  |  | R: GTGACCACCACACTCACTTA |
| Mt0-1-20Mb | I | 19555790 | F: CCTGTTTGGTGTTATCAAGTTGTG |
|  |  |  | R:ACCATATTCTCATTACCAAATTGG |
| Mt0-1-25Mb | I | 25193550 | F: TTTCTCACGTTGGCTTACAGC |
|  |  |  | R: ACAGGCATGTAAGTGCCAGG |
| spot3-13 | I | 27642471 | F: GCAGAGTGAAAAACCAGCATCC |
|  |  |  | R: TGGATACATGGAAGACTTGTCTCTC |
| F1B16-1 | I | 28261477 | F: TTTCCAAGAAAACTCACACT |
|  |  |  | R: GGTGGATATCGACTTAGG |
| Mt0-2-1Mb | II | 846640 | F: GGAAGCAGAGTCTTGTTGGTG |
|  |  |  | R: ACGGTTACGTAGAGTAAGCTATG |
| T17A11-1 | II | 5388871 | F: GAAGATACTTCCCAGACA |
|  |  |  | R: CCAACCACGCATTAACTA |
| T16B14-1 | II | 9416667 | F: ATGGCGGCTTGTTCCTTG |
|  |  |  | R: CCGCAGCATTAGCTAGTG |
| Mt0-2-13Mb | II | 12712150 | F: CGACCATGGTTCGGCTAG |
|  |  |  | R: GGAATTGGGATCATGGTTTCCTAC |
| F11F19-1 | II | 15100106 | F: GTACTGGATGTCAAACTAGA |
|  |  |  | R: ATAGCATGGTGATAAATAAG |
| 2-15.44 Mb | II | 15438817 | F: GAGCAATCCAGTAGAGGATA |
|  |  |  | R: CTTGAAGCTTAAATCTCAGC |
| 2-15.77 MB | II | 15769647 | F: GGCTCCTCCGAGCAATTA |
|  |  |  | R: CGCCCCCGCTCCCATTAG |
| 2-15.78 Mb | II | 15782813 | F: GAAGACGGATCACAAGCCTCTG |
|  |  |  | R: ATGGTGAGATGGGAAGAAGTGATG |
| 2-15.80 Mb | II | 15799459 | F: GACACATATGTAAAGCACTTAACTCC |
|  |  |  | R: CGAACCGTTAATGGATCTTCG |
| 2-15.81 Mb | II | 15816574 | F: GCGAGCTTTGCACAGACTTC |
|  |  |  | R: CGCCTTCTATACCAAACCGATC |
| 2-15.83 Mb | II | 15829129 | F: AATATCCTCACGGTAAAATG |
|  |  |  | R: GGTTAAATGAAACAATTTAG |
| 2-15.87 Mb | II | 15869896 | F: CGTAGATCTGACGTTCTCTGC |
|  |  |  | R: ATGATCGGAATTAACCTCTCCTG |
| 2-15.89 Mb | II | 15891414 | F: CGGAGAAAATTGCTATGACTGC |
|  |  |  | R: CTCTCCGACGCTATACCAC |
| 2-15.99 Mb | II | 15988203 | F: CGCCGTGAAATTAAAATG |
|  |  |  | R: GCTTAATCTCTGCTTATTTG |
| 2-16.20 MB | II | 16203906 | F: GTAGTAACGCTTTAGGCATA |
|  |  |  | R: TCACGTACTTGAGAAATACA |
| T16B24-1 | II | 16339214 | F: ATGAACGGAGTAGCTATC |
|  |  |  | R: CGCGTAGAACATAATCTGTA |
| F13H10-1 | II | 17235779 | F: TGTGTGAATCGTGATTGA |
|  |  |  | R: CACCACCATGTAAAACTTAG |
| F6E13-1 | II | 18261937 | F: CTAATATCAACTCGACTATC |
|  |  |  | R: TTGTTTTGTGCGAAAATC |
| 2-18.31 Mb | II | 18307762 | F: CCTTTAGCTAGCTGGTAGAAGAC |
|  |  |  | R: GCACATTAATTAAGAATGAAAACAGAG |
| 2-18.38 Mb | II | 18379490 | F: CGTGAATGCTTTAGGAGTCC |
|  |  |  | R: TGGATTCGTTGCATGATGCG |
| 2-18.39 Mb | II | 18395427 | F: AGAACTTATCGTCCCACGC |
|  |  |  | R: CTCCGCGCCCTTCATACC |
| 2-18.41 Mb | II | 18410897 | F: TGAAGGGGTCTATCTTTAATTATGGTC |
|  |  |  | R: GATTGTTTTTCTTTCATTGCAGCTG |
| 2-18.46 Mb | II | 18462450 | F: GGAGGTGATTCCGGTGTGGTAG |
|  |  |  | R: TGACAAAGCCGGATATGATGGC |
| 2-18.47 Mb | II | 18472092 | F: ATCTTCCCAAAACAGGAAACAAC |
|  |  |  | R: AAAATTGGCGCATTGTCTTGTG |
| 2-18.49 Mb | II | 18492177 | F: GCATTCTGCGTTCAACCTGC |
|  |  |  | R: CCCTAACCTGTTCTTTGGATC |
| 2-18.52 Mb | II | 18528032 | F: GGAGGTGTTTGTGCACAAGC |
|  |  |  | R: CCATATGGGAGACTAGTTCACTCC |
| 2-18.56 Mb | II | 18561850 | F: CCATTTTGCATTGAATCTTCATG |
|  |  |  | R: CCCATTAGAAACACTGATGGAC |
| 2-18.68 Mb | II | 18682317 | F: CGACAACAAATCTTTAGAAGAGTC |
|  |  |  | R: TGGGGATTGAAGATTTTAGAGATC |
| 2-18.79 Mb | II | 18797987 | F: CGGATTAATCTTGTCTTTGTTAG |
|  |  |  | R: TTAGTAGAGTGTGTGAGCGTA |
| T3F17-2 | II | 18956081 | F: GGCAAGAAATGTTCAAAG |
|  |  |  | R: GTAAGCCTGGTTGTGGAACT |
| 2-19.31 Mb | II | 19311525 | F: CCCGTACATTTTCTGCCAATG |
|  |  |  | R: GTTTCAGCGCTGATGCAAAGG |
| T3D7-1 | II | 19364303 | F: GGTATCGATTGAGCAAATAA |
|  |  |  | R: ACATGCGTCTGCTTGGAG |
| Mt0-2-19Mb | II | 19586280 | F: GTCTCCACTACGGTTCATTATCC |
|  |  |  | R: TTCGCGTCATATACACAACACAG |
| MXL8-1 | III | 7441951 | F: GTAGCCCAAAGCCGTACAG |
|  |  |  | R: GAGATGCGTTTCACCTACAA |
| F14O13-1 | III | 8624761 | F: TCAAAAAGCTAAACGATACA |
|  |  |  | R: GGCGATTATAGAGAAACAGA |
| Mt0-3-9Mb | III | 9194015 | F: CGAGACTATAGGAAGTCAAATATGAG |
|  |  |  | R: GCTTTGAGAGCCCTGACTTAC |
| T22B15-1 | III | 12774240 | F: AATTGGGCCAGCTTTGTTTC |
|  |  |  | R: GGCGGATCCACATGTATTGA |
| Mt0-3-22Mb | III | 22024220 | F: GTTTGACTATATCGACGGCAC |
|  |  |  | R: TGGTAATTAACCTCTGCTCG |
| Mt0-4-1Mb | IV | 1094800 | F: TGGTTGATCGTTTGATAAATTGTG |
|  |  |  | R: GTGATCGATTAGATGATGGTGAAC |
| Mt0-4-6Mb | IV | 5742320 | F: GCTTCGTCCCCAGTCATC |
|  |  |  | R: GCAAAAGTCATTACGGACAATACC |
| FCA2-1 | IV | 8610206 | F: TCAAGCGGACATATCAATAA |
|  |  |  | R: CCTCGGTCTACCATACAA |
| FCA8-1 | IV | 9791410 | F: TTCGGAGAAAGAAACGACAT |
|  |  |  | R: ATGGAACTATTCAGGCATTA |
| F18F4-1 | IV | 10871998 | F: TGCGTTTACACAAGACAACA |
|  |  |  | R: CATCTATACGCGGAACTCG |
| Mt0-4-16Mb | IV | 16720746 | F: ATCAACTTCGTTAGCATGAGACG |
|  |  |  | R: TTGCAATCCTTGGTAGCTTCAC |
| Mt0-4-18Mb | IV | 18069660 | F: GACAAAGAAAGAGCAGAGGAC |
|  |  |  | R: TGTTGCAGGCAGATAGAAAATTC |
| Mt0-5-0Mb | V | 182720 | F: GATGGGTTTTCCAGCTCCTG |
|  |  |  | R: CCACCCACTATAACCTTAGGT |
| Mt0-5-3Mb | V | 2611773 | F: GCGAATAAGAGGTTAGTGTGAC |
|  |  |  | R: GTTAAGGATAGGTCGGTTTGTG |
| Mt0-5-8Mb | V | 8219480 | F: TACTCAGTTAGGCGTATGTTATTCG |
|  |  |  | R: GCATGCATGGAACATAACCTC |
| Mt0-5-15Mb | V | 15443360 | F: ATTTGTGAGCTGTGAGTTGC |
|  |  |  | R: AACTCACGGCAAATCCAGAG |
| Mt0-5-20Mb | V | 19831990 | F: TGTTTCTACAATTGTGGTCCACC |
|  |  |  | R: GGTCTGTGATAATCCGATGAGG |

**Supplementary File 1C.** Mutants and transgenic lines used in this study.

| **Species** | **Name** | **Source** | **Identifier** |
| --- | --- | --- | --- |
| *Arabidopsis* *thaliana* | *mcr-1* | This study, EMS-mutagenesis | N/A |
| *Arabidopsis* *thaliana* | *mcr-2* | This study, EMS-mutagenesis | N/A |
| *Arabidopsis* *thaliana* | *mcr-3* | This study, EMS-mutagenesis | N/A |
| *Arabidopsis* *thaliana* | *mcr-4* | This study, EMS-mutagenesis | N/A |
| *Arabidopsis* *thaliana* | *mcr-5* | ABRC | CS853233 |
| *Arabidopsis* *thaliana* | *mcr-6* | ABRC | SALK_205528C |
| *Arabidopsis* *thaliana* | *mcr-7* | ABRC | SALK_203827C |
| *Arabidopsis* *thaliana* | *elmod_c* | ABRC | SALK_076565 |
| *Arabidopsis* *thaliana* | *elmod_d* | ABRC | SALK_031512 |
| *Arabidopsis* *thaliana* | *elmod_e* | ABRC | SALK_082496 |
| *Arabidopsis* *thaliana* | *elmod_f* | ABRC | SALK_010379 |
| *Arabidopsis* *thaliana* | *inp1-1* | (Dobritsa and Coerper, 2012) | N/A |
| *Arabidopsis* *thaliana* | *mcr-1* *inp1-1* | This study, genetic cross | N/A |
| *Arabidopsis* *thaliana* | *inp2-1* | (Lee et al., 2021) | N/A |
| *Arabidopsis* *thaliana* | *mcr-1* *inp2-1* | This study, genetic cross | N/A |
| *Arabidopsis* *thaliana* | *d6pkl3-2* | (Lee et al., 2018) | N/A |
| *Arabidopsis* *thaliana* | *mcr-1* *d6pkl3-2* | This study, genetic cross | N/A |
| *Arabidopsis* *thaliana* | *tes* | ABRC | SALK_113909 |
| *Arabidopsis* *thaliana* | *mcr-1* *tes* | This study, genetic cross | N/A |
| *Arabidopsis* *thaliana* | *MiMe* | (d’Erfurth et al., 2009) | N/A |
| *Arabidopsis* *thaliana* | *mcr-1* *MiMe* | This study, genetic cross | N/A |
| *Arabidopsis* *thaliana* | *cenh3 GFP-tailswap* | (Ravi and Chan, 2010), ABRC | CS66982 |
| *Arabidopsis* *thaliana* | *elmod_a* | This study, CRISPR/Cas9 | N/A |
| *Arabidopsis* *thaliana* | *mcr-1 elmod_a* | This study, genetic cross | N/A |
| *Arabidopsis* *thaliana* | *elmod_e^CR^* | This study, CRISPR/Cas9 | N/A |
| *Arabidopsis* *thaliana* | *mcr-1 elmod_ e^CR^* | This study, CRISPR/Cas9 | N/A |
| *Arabidopsis* *thaliana* | *mcr-1 elmod_ c* | This study, genetic cross | N/A |
| *Arabidopsis* *thaliana* | *mcr-1 elmod_ d* | This study, genetic cross | N/A |
| *Arabidopsis* *thaliana* | *mcr-1 elmod_ f* | This study, genetic cross | N/A |
| *Arabidopsis* *thaliana* | *inp1-1 DMC1pr:INP1-YFP* | (Dobritsa et al., 2018) | N/A |
| *Arabidopsis* *thaliana* | *mcr-1 DMC1pr:INP1-YFP* | This study, genetic cross | N/A |
| *Arabidopsis* *thaliana* | *d6pkl3-2 D6PKL3pr:D6PKL3-YFP* | (Lee et al., 2018) | N/A |
| *Arabidopsis* *thaliana* | *mcr-1 D6PKL3pr:D6PKL3-YFP* | This study, genetic cross | N/A |
| *Arabidopsis* *thaliana* | *mcr-1 MCRpr:gMCR* | This study, plant transformation |  |
| *Arabidopsis* *thaliana* | *mcr-1 MCRpr:gMCR-YFP* | This study, plant transformation | N/A |
| *Arabidopsis* *thaliana* | *mcr-1 MCRpr:MCR CDS* | This study, plant transformation | N/A |
| *Arabidopsis* *thaliana* | *mcr-1 elmod_a EApr:gELMOD_A* | This study, plant transformation | N/A |
| *Arabidopsis* *thaliana* | *mcr-1 elmod_a EApr:gELMOD_A-YFP* | This study, plant transformation | N/A |
| *Arabidopsis* *thaliana* | *mcr-1 EApr:gELMOD_A-YFP* | This study, plant transformation | N/A |
| *Arabidopsis* *thaliana* | *mcr-1 MCRpr:gELMOD_A-YFP* | This study, plant transformation | N/A |
| *Arabidopsis* *thaliana* | *mcr-1 MCRpr:gELMOD_C-YFP* | This study, plant transformation | N/A |
| *Arabidopsis* *thaliana* | *mcr-1 MCRpr:gELMOD_D-YFP* | This study, plant transformation | N/A |
| *Arabidopsis* *thaliana* | *mcr-1 MCRpr:gELMOD_E-YFP* | This study, plant transformation | N/A |
| *Arabidopsis* *thaliana* | *mcr-1 MCRpr:gELMOD_F-YFP* | This study, plant transformation | N/A |
| *Arabidopsis* *thaliana* | *mcr-1 ELMOD_Epr:gELMOD_E-YFP* | This study, plant transformation | N/A |
| *Arabidopsis* *thaliana* | Col-0 *ELMOD_Epr:gELMOD_E-YFP* | This study, plant transformation | N/A |
| *Arabidopsis* *thaliana* | *mcr-1 MCRpr:gMCR^R127K^-YFP* | This study, plant transformation | N/A |
| *Arabidopsis* *thaliana* | *mcr-1 MCRpr:gMCR ^D121N^-YFP* | This study, plant transformation | N/A |
| *Arabidopsis* *thaliana* | *mcr-1 MCRpr:gMCR ^G129C^-YFP* | This study, plant transformation | N/A |
| *Arabidopsis* *thaliana* | *mcr-1 MCRpr:gMCR ^D121N/G129C^-YFP* | This study, plant transformation | N/A |
| *Arabidopsis* *thaliana* | *mcr-1 elmod_a EApr:gELMOD_A^R127K^-YFP* | This study, plant transformation | N/A |
| *Arabidopsis* *thaliana* | *mcr-1 MCRpr:gELMOD_E ^N121D^-YFP* | This study, plant transformation | N/A |
| *Arabidopsis* *thaliana* | *mcr-1 MCRpr:gELMOD_E ^C129G^-YFP* | This study, plant transformation | N/A |
| *Arabidopsis* *thaliana* | *mcr-1 MCRpr:gELMOD_E ^N121D/C129G^-YFP* | This study, plant transformation | N/A |
| *Arabidopsis* *thaliana* | Col-0 *MCRpr:H2B-YFP* | This study, plant transformation | N/A |
| *Arabidopsis* *thaliana* | Col-0 *EApr:H2B-YFP* | This study, plant transformation | N/A |
| *Arabidopsis* *thaliana* | *mcr-1 MCRpr:gMCR-YFP MCRpr:gELMOD_E-YFP* | This study, genetic cross | N/A |
